# Isodose surface differences: A novel tool for the comparison of dose distributions

**DOI:** 10.1002/acm2.14085

**Published:** 2023-10-04

**Authors:** Stefanos Diamantopoulos, Kalliopi Platoni, Pantelis Karaiskos, Vassilis Kouloulias, Efstathios Efstathopoulos

**Affiliations:** ^1^ 2nd Department of Radiology University General Hospital “Attikon” 1 Rimini Street National and Kapodistrian University of Athens Chaidari Greece; ^2^ Medical Physics Laboratory Medical School National and Kapodistrian University of Athens Athens Greece; ^3^ Joint Department of Physics The Royal Marsden NHS Foundation Trust London UK

**Keywords:** dose measurement, PSQA, Quality Assurance, IMRT/VMAT

## Abstract

**Background:**

Comparing dose distributions is a routine task in radiotherapy, mainly in patient‐specific quality assurance (PSQA). Currently, the evaluation of the dose distributions is being performed mainly with statistical methods, which could underestimate the clinical importance of the spotted differences, as per the literature.

**Purpose:**

This paper aims to provide proof‐of‐concept for a novel dose distribution comparison method based on the difference of the isodose surfaces. The new method connects acceptance tolerance to QA limitations (equipment capabilities) and integrates a clinical approach into the analysis procedure.

**Methods:**

The distance of dose points from the isocenter can be used as a function to define the shape of an isodose surface expressed as a histogram. Isodose surface differences (ISD) are defined as the normalized differences of reference and evaluated surface histograms plotted against their corresponding isodose. Acceptance tolerances originate from actual QA tolerances and are presented clinically intuitively. The ISD method was compared to the gamma index using intentionally erroneous VMAT and IMRT plans.

**Results:**

Results revealed that the ISD method is sensitive to all errors induced in the plans. Discrepancies are presented per isodose, enabling the evaluation of the plan in two regions representing PTV and Normal Tissue. ISD manages to flag errors that would remain undetected under the gamma analysis.

**Conclusion:**

The ISD method is a meaningful, QA‐related, registration‐free, and clinically oriented technique of dose distribution evaluation. This method can be used either as a standalone or an auxiliary tool to the well‐established evaluation procedures, overcoming significant limitations reported in the literature.

## 1 INTRODUCTION

The comparison of two dose distributions is a routinely performed task in radiation therapy, mainly in patient‐specific quality assurance (PSQA). Traditionally, dose comparison results have been analyzed using statistical methods, with the gamma index serving as the gold standard.^1^, ^2^

This index combines two indices (dose difference and distance‐to‐agreement‐DTA) to a unit‐independent quantity. The acceptance of dose distribution is based on the percentage of measured points meeting either the dose or distance criteria, which can vary depending on the planning technique.^3^ However, the gamma index has been criticized for concealing discrepancies due to certain intrinsic limitations.^4^, ^5^, ^6^, ^7^, ^8^, ^9^, ^10^, ^11^, ^12^ Numerous indices or variants of the original gamma method have been proposed to account for the deficiencies mentioned above.^13^ Nonetheless, an “ideal” evaluation index^14^ has yet to be designed due to physical and measurement limitations.

Alternative approaches can overcome some of the limitations of statistical methods. Dose comparison in an “isodose per isodose” mode could lead to a more intuitive understanding of the evaluated dose discrepancies, imitating the treatment planning assessment process. This methodology is well recognized by medical physicists, as it was the fundamental qualitative evaluation of 2D distributions in the early days of IMRT.^15^ Nevertheless, reliable quantification of the results in three‐dimensional space should be established.

A pair of three‐dimensional objects can be compared with various methods, some of which have been developed in the context of pattern recognition. The majority of them are time‐consuming, as complex mathematical procedures are required. A method to compare 3D objects based on histogram dissimilarities was introduced in 2002,^16^ which offers a more practical approach to this problem. It is possible to apply the “shape distributions” method, which can provide shape signatures for 3D objects, to the verification process under the appropriate modifications and adjustments to radiotherapy concepts. A pair of 3D isodoses/shells can be assessed by geometrically parametrizing them into histograms, avoiding any calculations on their initial irregular shape.

This paper aims to provide proof‐of‐concept for development and testing a novel method for evaluating the dose distributions based on the geometrical distance of isodose surfaces. The proposed “Isodose Surface Differences” (ISD) method is a distance‐based isodose shape comparison tool that offers a more clinically intuitive approach to the verification analysis procedure. We define ISD as a graphical diagram of isodose‐shape discrepancies over the whole range of a plan. Tolerance criteria are defined per plan, individually for each dose level, following the local dose gradient while being automatically generated from a common starting point: the mechanical performance limits of the collimating device of the linac. The performance of the ISD method was investigated with intentionally introduced errors in various treatment plans and compared against the gamma index.

## 2 METHOD

The Isodose Surface Differences (ISD) method compares dose distributions according to the shape of their isodoses in space. A detailed description of the critical components of this method is presented in the following paragraphs.

### 2.1 Isodose surface histogram (his_x_)

An isodose curve can be considered a three‐dimensional closed surface or shell consisting of n points $p_{x}\left(n\right)$ (or voxels on a regular 3D dose grid) with the same dose value *x*. Other shells with lower dose values enclose each isodose surface $is_{x}$, while it contains shells with higher dose values. Therefore, a planned dose distribution DD (Equation 1) is the set of all isodose surfaces with dose values from 0 to D_max_ (Gy).

**FIGURE 1:**
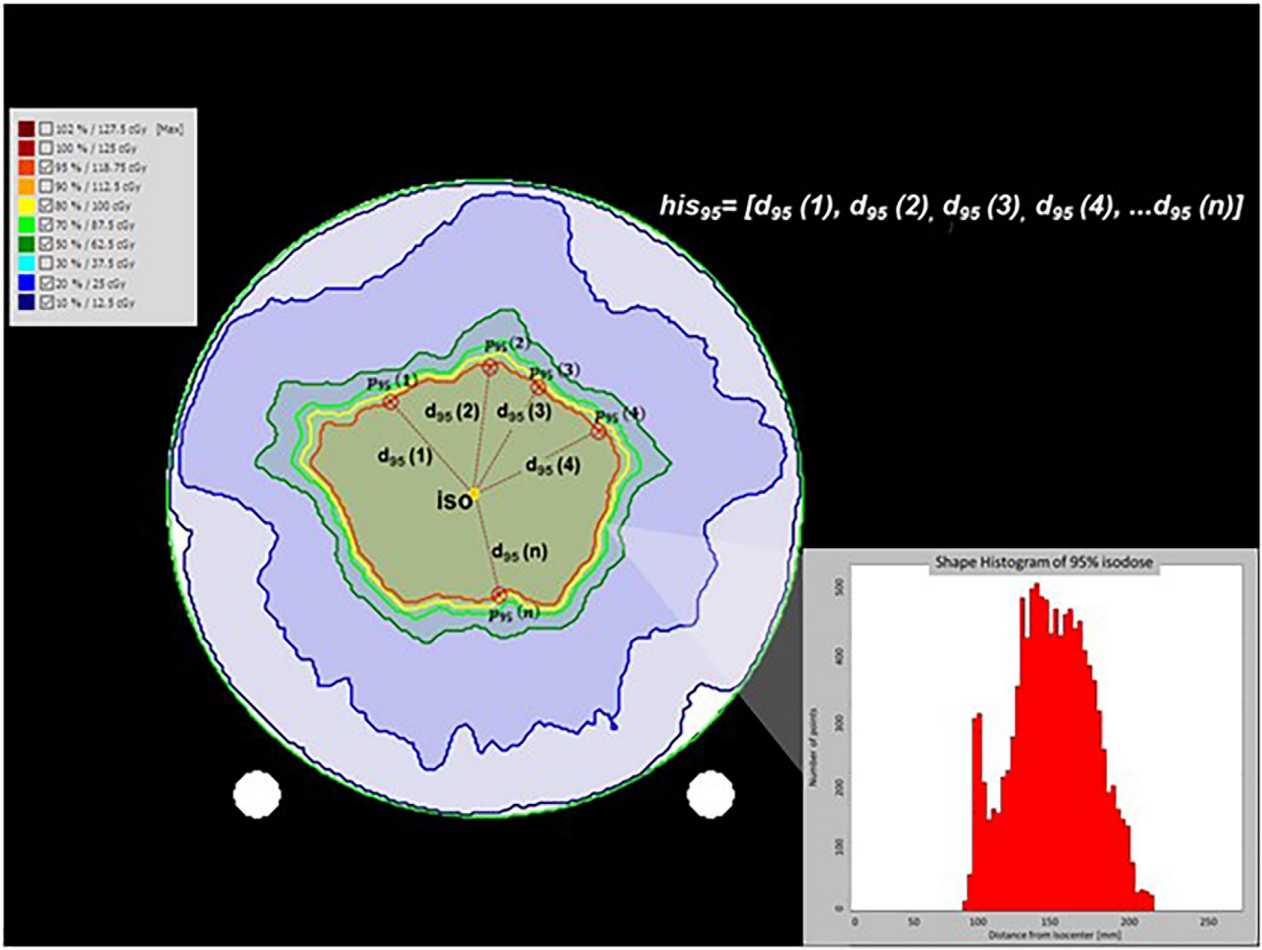
For illustration purposes, we schematically represent the construction of an Isodose Surface Histogram ($\mathrm{hi}s_{x}$) from the distances of the points of a selected isodose from the isocenter (iso). For the sake of simplicity, a 2D example is presented. The exact process is performed in 3D space. In this case, the 95% isodose of a prostate verification plan is converted to the red‐colored histogram.

The Isodose Surface Histogram ($his_{x}$) is the histogram that emerges from the geometrical properties of an isodose surface $is_{x}$ (Equation 2). The measurement of the Euclidean distance of isodose surface points $p_{x}\left(n\right)$ from the isocenter (iso) of the plan produces the ISH histogram (Figure 1).

(1) $$his_{x}=\sum_{i=1}^{bins_{x}}d_{x}{\left(n\right)}_{i}$$

Where,

(2) $$d_{x}\left(n\right)=\sqrt{\sum_{i=1}^{3}{\left[p_{x}{\left(n\right)}_{i}-iso\right]}^{2}}$$

& bins_x_ is defined by using Doane's Formula.^17^

### 2.2 Comparison of his_x_

The total dose distribution can be evaluated by comparing the shape of each isodose from the evaluation plan with the corresponding isodose from the reference plan. A pairwise comparison of the evaluation histogram $his_{x}\left(eval\right)$ versus the reference one, $his_{x}\left(ref\right)$ simplifies the complex three‐dimensional assessment of the shape of an isodose x. The latter is performed via the Bray‐Curtis dissimilarity measure.^18^

(3) $$BC_{x}=\frac{\sum_{i}\left|his_{x}{\left(eval\right)}_{i}-his_{x}{\left(ref\right)}_{i}\right|}{\sum_{i}\left(his_{x}\left(eval\right){\;}_{i}+his_{x}{\left(ref\right)}_{i}\right)}$$

Where $BC_{x}$ is the Bray‐Curtis dissimilarity value for the isodose level x, and i is the i‐th bin. The $BC_{x}$ value can range from 0 to 1, with the zero‐value meaning a perfect match. The discrepancies in the entire plan can be visualized by plotting the function $BC_{x}$ against their respective isodose levels x. Furthermore, underdosage or overdosage can be detected by comparing the median values of the evaluation and reference histograms, $\tilde{his_{x}}\;\left(eval\right)$ and $\tilde{his_{x}}\;\left(ref\right)$ respectively. The condition where $\tilde{his_{x}}\;\left(eval\right)$> $\;\tilde{his_{x}}\;\left(ref\right)$ indicates that the isodose surface is located further from the isocenter; therefore, the evaluation plan is “hotter” and vice versa.

### 2.3 Definition of a tolerance area

Globally applied acceptance limits are not suitable for isodose comparison. Instead, each isodose surface should be evaluated separately, according to the regional dose gradient. Therefore, a 3D acceptance area should be formed individually around each evaluated isodose surface before comparing the dose distributions. In the reference distribution, the radius of an equivalent sphere is calculated for each isodose volume. Then, the radius difference between successive isodoses (e.g., 1% increments), which can be used as a measure of the regional gradient of the plan,^19^ is plotted against the various isodose levels. From the derived graph, the midrange of the maximum and minimum radius difference is calculated. The isodose where the midrange corresponds is selected as the reference isodose (ISO_mr_). Consequently, the ISO_low_ and ISO_up_ limits are defined as the isodoses that are located ± 1 mm apart from ISO_mr_, respectively (same spatial criterion as for MLC QA.^20^, ^21^) ISO_up_ is the limit toward a higher valued isodose, and ISO_low_ is the limit toward a lower one. Therefore, ISO_up_ would always be located closer to the isocenter. Then, the fixed‐dose interval (ISO_up_‐ISO_low_) is universally applied in each isodose of the reference plan resulting in a non‐uniform spatial criterion.

**FIGURE 2:**
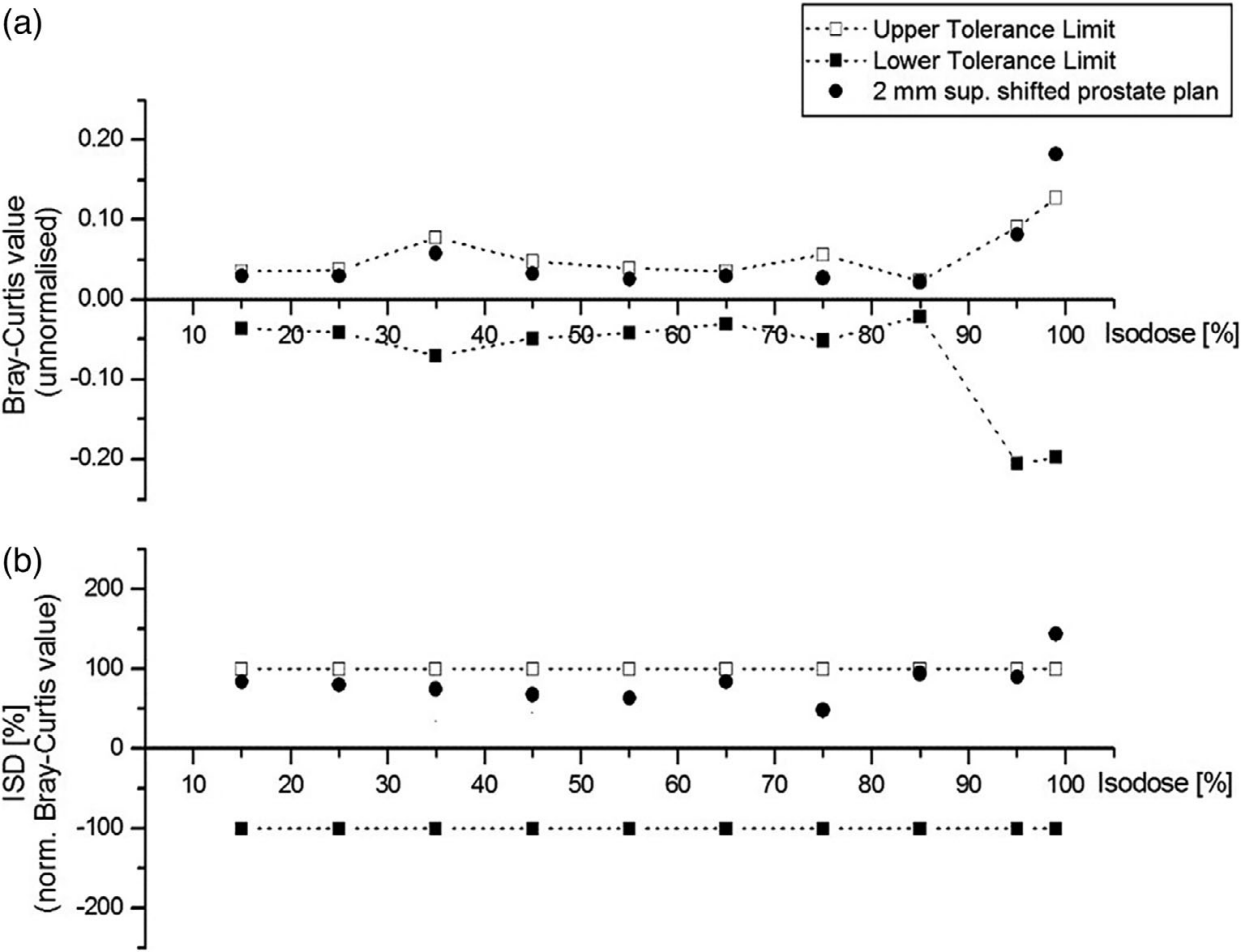
(a) Un‐normalized (raw) isodose surface differences and their respective tolerances, expressed as Bray‐Curtis values and (b) normalized isodose surface differences (ISD) and their respective tolerances, expressed as percentages for the same prostate plan, shifted superiorly by 2 mm.

Finally, each isodose histogram is compared with its respective ISO_low_ and ISO_up_ histograms, defining individual acceptance zones expressed in BC dissimilarity values,$\;BC_{ISO_{low}}$ and $BC_{ISO_{up}}$, respectively. Lower and upper limits are not necessarily symmetrical, as they emerge from two different histogram comparisons (Figure 2).

### 2.4 Isodose surface difference graphs

For optimal visualization of the discrepancies against tolerance limits, we convert the raw comparison data (Figure 2a) to a more comprehensible format. Isodose Surface Difference (ISD) is the Bray‐Curtis (BC) value calculated from the comparison of the evaluation histogram with the reference one, normalized to the respective acceptance limit for a specific isodose (Figure 2b). Two limits (upper and lower) exist per isodose; therefore, we use the following formula for normalization:

(4) $$ISD\;\left[\%\right]=\left\{\begin{array}{c} \frac{BC_{x}\;}{\;BC_{ISO_{up}}}\times 100,\;{\tilde{his}}_{x}\left(eval\right)\geq \;{\tilde{his}}_{x}\left(ref\right)\\ -\frac{BC_{x}\;}{\;BC_{ISO_{low}}}\times 100,\;{\tilde{his}}_{x}\left(eval\right)<\;{\tilde{his}}_{x}\left(ref\right)\; \end{array}\right.$$

Conclusively, we create a triad of points (BC values) per isodose x: $his_{x}\;\left(eval\right)$ versus $his_{x}\;\left(ref\right)$ BC value, the upper limit $his_{\mathrm{ISOup}\;}\left(ref\right)$ versus $his_{x}\;\left(ref\right)$, and the lower limit$\;his_{\mathrm{ISOlow}}\;\left(ref\right)$ versus $his_{x}\;\left(ref\right)$. An ISD graph depicts all normalized isodose surface shape differences versus their corresponding isodose.

The x‐axis shows the isodose levels while the y‐axis represents ISD values expressed in percentage. A pair of parallel lines at the ± 100% axis position extends across the isodose range and indicates the upper and the lower limit of acceptance, respectively. The y‐axis expresses the deviation of the evaluated isodose from the reference isodose. An ISD value of 0% denoted that reference and evaluated isodose shapes are identical. An ISD value of 100% means that the evaluated isodose shape coincides with the upper tolerance isodose shape.

Tolerance lines are artificially adjusted to be straight and parallel to each other to be easily comprehensible. Continuous points neither create these lines nor do their points correlate. A separate pair of upper and lower limits for each isodose is calculated depending on the number of isodose points, voxel size, etc. In most cases, unmodified tolerance lines follow a rising trend with increasing isodose values. Nevertheless, the proposed modification does not distort the spotted discrepancies and the relative difference from their corresponding limits (Figure 2).

### 2.5 Verification of the ISD method

We evaluated the presented method by generating four VMAT treatment plans for four commonly treated sites (brain, head and neck, lung, and prostate) to account for differently shaped dose distributions. An additional breast case was also planned using a forward‐IMRT technique (two tangential fields). For all sites, a 6 MV photon beam model was used. The plans were created in the treatment planning system Eclipse version 15.1.52 (Varian Medical Systems, Palo Alto, California, US).

The five clinical plans were propagated on a cylindrical and homogenous water‐equivalent phantom (HU = 0), simulating a typical verification plan preparation process. Then, multidirectional positional errors (1 mm‐10 mm) were introduced in the plans. The shifts were gradually increased in steps of 1 mm and applied to each direction separately (longitudinal, lateral, and vertical). A set of combined multidirectional positional errors was also introduced. Every erroneous plan was recalculated with a similar dose grid to the reference one (1 × 1 × 1 mm^3^), resulting in distorted distributions on the phantom.

Initially, we compared all erroneous plans to the original (reference) verification plan. The gamma index method was used with a 3% dose difference and a 2 mm DTA, following the recommendation of AAPM Report No. 218,^2^ with a minimum acceptance rate of 95%.

Subsequently, we performed the comparison using the ISD method, which was automated in an in‐house Python 3.7.7. script. This script facilitates (a) the detection of selected isodose levels, (b) their transformation to shape histograms, (c) the generation of acceptance limits, and (d) the pairwise comparison of each reference isodose to the evaluated ones according to the principles mentioned above. The results are plotted in four ISD charts per site, accompanied by their respective gamma index passing rates. Furthermore, the mean absolute ISD value per comparison was plotted against the gamma index. The most typical cases were presented.

## 3 RESULTS

**FIGURE 3:**
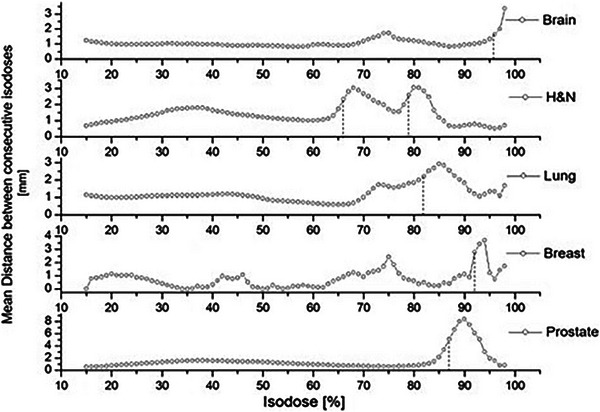
Mean distance between consecutive isodoses of 1% increments for the reference plans of the five sites. The vertical dashed lines indicate the place of the ISO_MR isodose.

In Figure 3, we present the mean distances between successive isodoses for the reference plans of the different anatomical sites. The range of distances and the variation per isodose level is different per site, which is distinctive for each plan. The prostate plan has the largest range of distances (with a maximum isodose distance of 8.3 mm). The maximum distance also varies per site. The head and neck plan, a simultaneous integrated boost plan with two dose prescription levels, has two local maxima at 69% and 81% isodose. The latter indicates the presence of the two targets.

Plotting the mean distance of isodoses reveals the dose threshold involved in PTV target coverage. Due to the different “anatomy” of the verification phantom, this threshold differs from the actual plan. The threshold isodose (ISO_mr_) for the brain plan is defined as 96%, for H&N as 79% (considering only the higher dose target), for the lung plan as 83%, for the breast as 92%, and the prostate as 87%.

Consequently, according to the local gradient of these isodose levels, the spatial criterion of ± 1 mm will correspond to ± 0.4% (brain), ± 0.3% (H&N), ± 0.3% (lung), ± 0.2% (breast), and ± 0.3% (prostate) dose difference (ISO_up_ & ISO_low_). We generated the final ISD plots based on these values and following the described methodology.

### 3.1 ISD chart description

We present the full results of this dose comparison approach in the Appendix (Figures A9 to A13) in the form of ISD charts of the different treatment sites. Here, we report an indicative set of results for one case and one set of induced errors, accompanied by the values of the corresponding gamma passing rate. The rest of the cases have similar behavior.

The range of isodoses is divided into two major regions: “Normal Tissue” and “PTV.” We define The PTV (dashed line onwards) as the isodose range above the ISO_mr_. Beyond this point, the isodoses conform to the target. The phantom has a different morphology than an actual patient; therefore, we expect a different isodose than the 95% (for typically fractionated treatments) to be accounted for target coverage. The rest of the isodoses (“Normal Tissue” region) contribute the most to the organ‐at‐risk dose. This division enables further clinical insight into the behavior of the evaluated plan.

### 3.2 Comparison of ISD with gamma index

**FIGURE 4:**
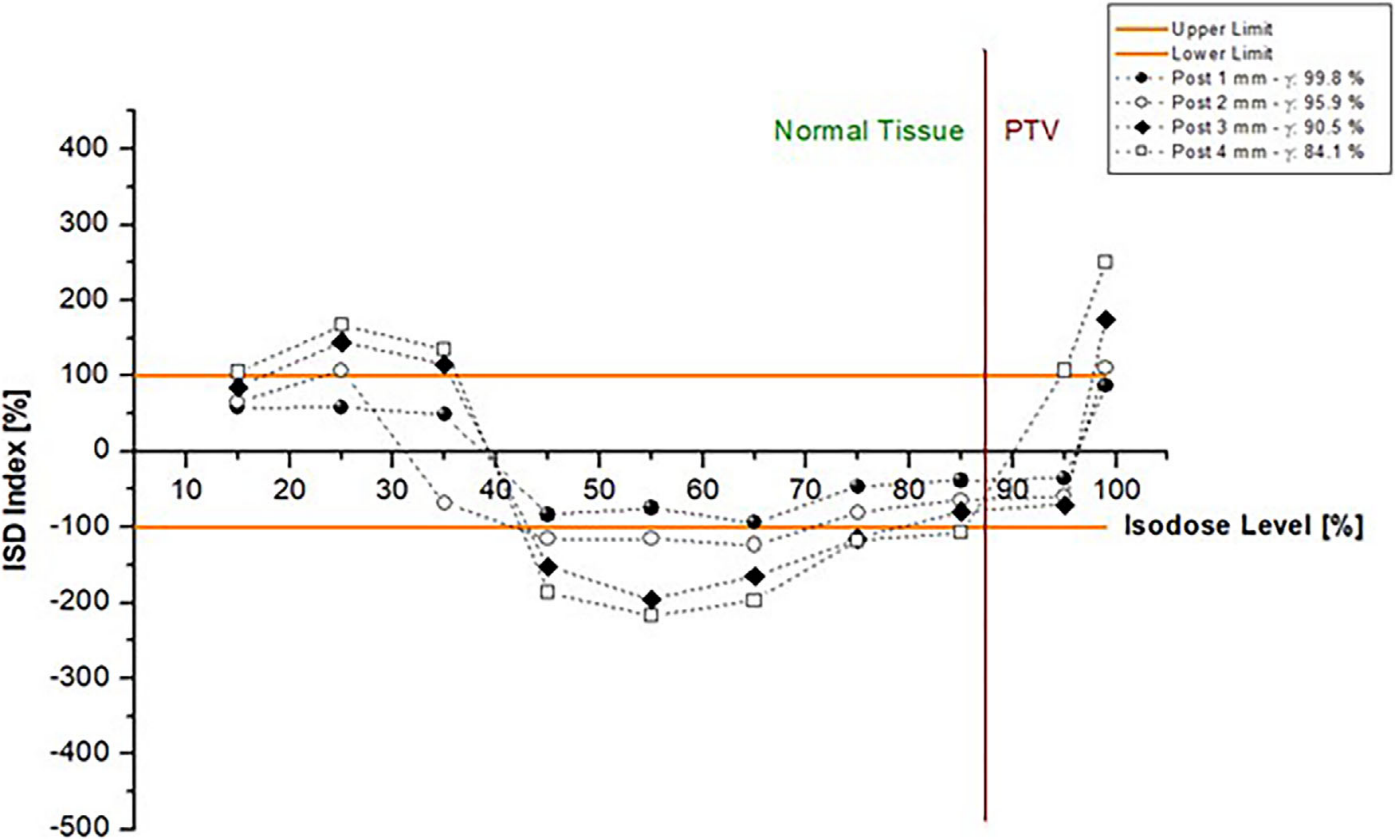
ISD graphs and gamma index passing rates (3%/2 mm) for the prostate plans shifted posteriorly compared against the reference plan. All shifts are in mm.

**FIGURE 5:**
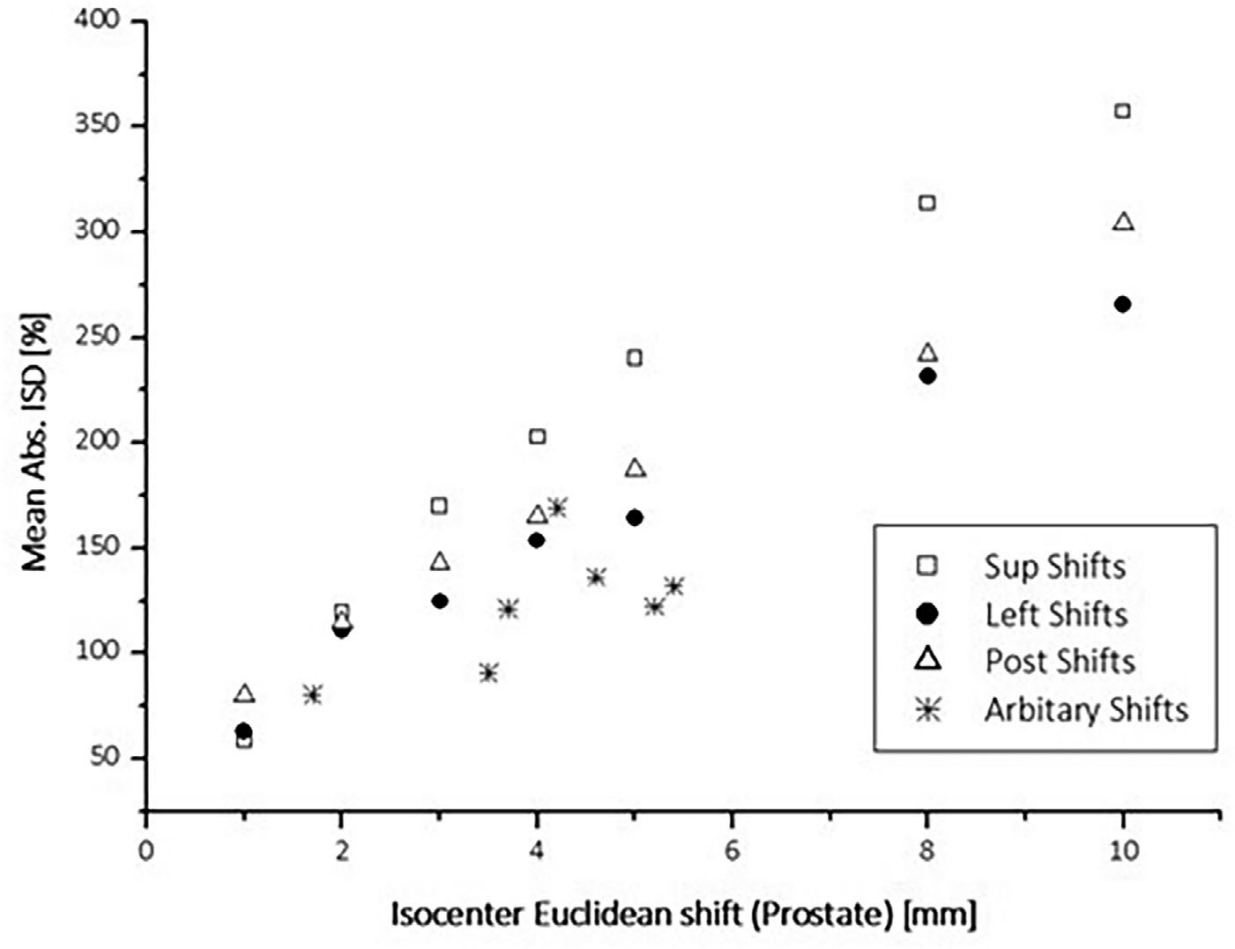
Mean absolute ISD value for all distorted prostate plans in relation to the applied shifts.

Figure 4 demonstrates the results for posteriorly shifted prostate plans compared to the reference plan. With the increasing magnitude of the induced shifts, the ISD chart presents increasing deviations from zero. The latter is further supported by Figure 5, where we present the mean absolute ISD value for all distorted prostate plans versus the applied shifts. It is observed that the direction of the shift can be distinguished, as the single directional errors follow a different slope.

### 3.3 Case‐by‐case analysis

#### 3.3.1 Brain

**FIGURE 6:**
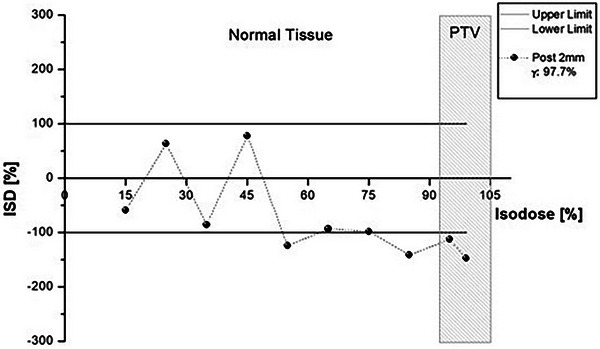
ISD and gamma (3%/2 mm) comparison of the reference plan with the 2 mm posteriorly shifted brain plan.

For the 2 mm post shift of the brain plan, the gamma index of 3%/2 mm gave a passing rate of 97.7%, while the ISD method indicated differences from the intermediate doses (55%) up to the 100% isodose (Figure 6). Inspection of the ISD plot indicates that the evaluated plan is cooler in this dose region.

#### 3.3.2 Head and neck

**FIGURE 7:**
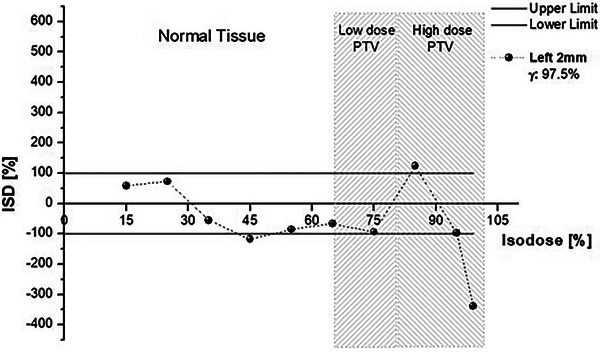
ISD and gamma (3%/2 mm) comparison of the reference plan with the 2 mm laterally shifted H&N plan.

The lateral shift of the isocenter by 2 mm resulted in an acceptable plan according to gamma analysis (97.5%). The ISD method, however, indicated isodoses out‐of‐tolerance for this shift, both in the healthy tissue area (45%) and in the target area (85% and 100%), as shown in Figure 7. Moreover, for the same shift, the ISD method spotted some isodoses on the acceptance limits (e.g., 75% and 95%).

#### 3.3.3 Prostate

**FIGURE 8:**
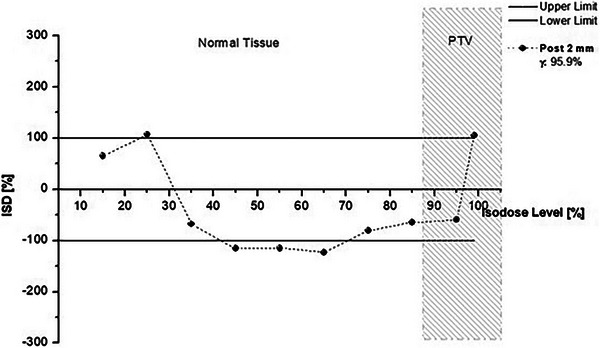
ISD and gamma (3%/2 mm) comparison of the reference plan with the 2 mm posteriorly shifted prostate plan.

The 2 mm posteriorly shifted prostate plan was considered acceptable with the gamma method (passing rate 95.9%). However, the ISD method identified half of the checked isodoses out of limits (Figure 8).

## 4 DISCUSSION

The ISD method proved to be sensitive in detecting every shift. Even for induced errors that allow the shape of the isodoses to remain within limits, there is a clear indication of the mismatch, a non‐zero ISD value, that the user should be aware of. In addition, errors of increased severity caused a linear increase in ISD values, a desired property of a dose comparison index.

**FIGURE A1:**
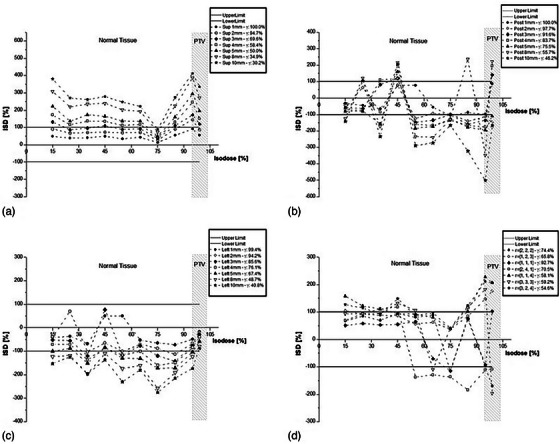
ISD graphs and gamma index passing rates (3%/2 mm) for the brain plans shifted (a) superiorly, (b) posteriorly, (c) left, (d) in all three directions compared against the reference plan. All shifts are in mm.

**FIGURE A2:**
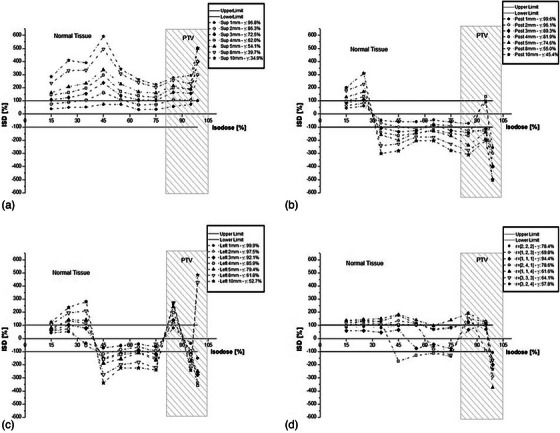
ISD graphs and gamma index passing rates (3%/2 mm) for the head and neck plans shifted (a) superiorly, (b) posteriorly, (c) left, (d) in all three directions compared against the reference plan. All shifts are in mm.

As shown in the Appendix, ISD appears to be following gamma index passing rate results; the gamma index fails, and at least one of the ISD points is out of tolerance. It is pertinent to note that the reverse is not observed (passed ISD and failed gamma index). For the brain case (Figure A1), ISD detected that a 2 mm vertical shift would significantly impact plan validity at the high‐dose region. Under the gamma analysis, the plan would pass with a 97.7% passing rate. The head and neck plan (Figure A2) revealed a similar behavior between the ISD method and the gamma index. In both 2 mm posteriorly and 2 mm left‐shifted distributions, the ISD method showed differences that would be considered within limits using gamma with 96.1% and 97.7% passing rates, respectively. For the first case, the acceptance of that plan would probably result in “cooler” higher doses inside the PTV than the reference one, as the 95% isodose ISD value is much lower than the tolerance limit.

**FIGURE A3:**
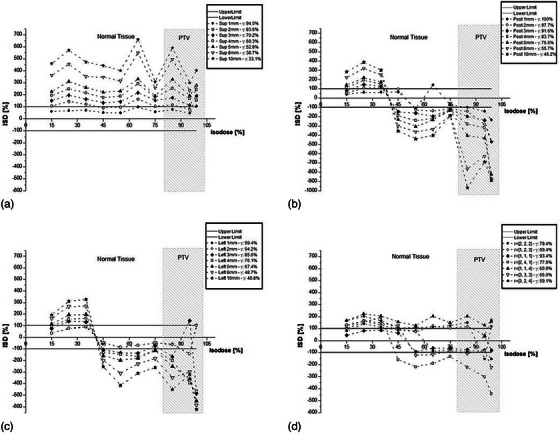
ISD graphs and gamma index passing rates (3%/2 mm) for the lung plans shifted (a) superiorly, (b) posteriorly, (c) left, (d) in all three directions compared against the reference plan. All shifts are in mm.

**FIGURE A4:**
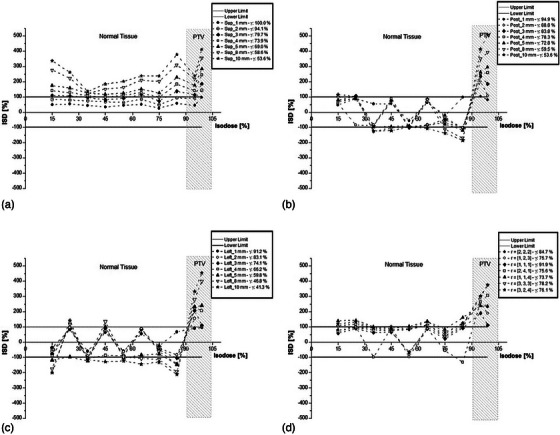
ISD graphs and gamma index passing rates (3%/2 mm) for the breast plans shifted (a) superiorly, (b) posteriorly, (c) left, (d) in all three directions compared against the reference plan. All shifts are in mm.

**FIGURE A5:**
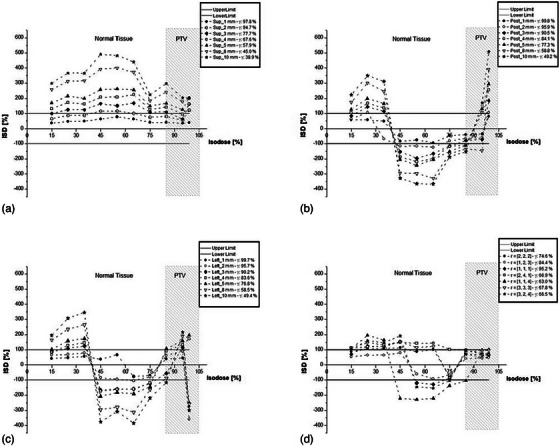
ISD graphs and gamma index passing rates (3%/2 mm) for the prostate plans shifted (a) superiorly, (b) posteriorly, (c) left, (d) in all three directions compared against the reference plan. All shifts are in mm.

The difference in shapes is also reflected in the difference in volume; the evaluated 95% isodose volume is reduced by 9% compared to the reference. The “failed” 45% isodose for the lateral shift would benefit the patient, as the dose would be lower than expected in the “Normal Tissue” region. In the PTV area, the failing 85% isodose would benefit the patient as it contributes more dose to the PTV. In contrast, the 95% and 99% isodoses would have opposite effects. A similar pattern appeared for the lung (Figure A3) and breast (Figure A4) plans. All the gamma index failed plans also failed under ISD analysis. According to the ISD method, the vertical shifts of 1 and 2 mm and the lateral shifts of 1 mm fail at some isodose levels. However, they passed under the gamma index. For the prostate plans (Figure A5), the ISD and gamma disagreement appeared not only in the one‐directional shifted plans but also at the composite 1 mm superior, 1 mm posterior, and 1 mm left shift, where the gamma method “accepted” this plan and ISD showed under dosage to the PTV dose range.

ISD is a newly introduced idea. Therefore, we adopted the most straightforward approach during its development to facilitate the comprehension of the properties and behaviors of the method. Nevertheless, further improvements are feasible to refine the method according to radiation therapy concepts.

Shape signature histograms can be generated in a variety of ways. The original shape distribution methodology^16^ suggests that several geometric properties of a 3D object (the isodose surface in this study) could be used for this purpose. An appropriate isodose histogram generation condition could be, for instance, the distance between two randomly selected points on the isodose surface, the area formed by three points, or even the volume defined by four points on the surface. More complex functions could be devised as well.

In this study, the Euclidean transformation is used, which is the Euclidean distance of the surface points from a fixed point in space. The key advantages of this decision are the following. Firstly, the isocenter is a reference point in radiotherapy, suitable for this proof of principle. Using the isocenter as a parametrization point in ISD methodology eliminates the need for a “perfect” registration between the reference and evaluated distributions, as only the registration of isocenters’ coordinates is required. According to the literature, the majority of the existing methods require an accurate registration of the distribution in order to produce valid results.^22^

Second, the “median distance from the isocenter” of the evaluated isodoses (retrieved from the histograms’ median) designates whether the points are located further from or closer to the isocenter than expected. This information allows using a positive or a negative sign to describe the differences by indicating whether an error causes over or under‐treatment across the isodose range. This property forms a deeper understanding of each discrepancy.

To extract this information robustly, the parametrization point (isocenter) should be located centrally in the distribution, although the changes in the shape of the isodoses are detectable regardless. If the isocenter is located outside the high dose area, a relevant point in the middle of the distribution should be selected instead. For example, any dose specification point could serve this purpose, even the coordinates of the central detector of the verification device. Finally, the number of isocenters in a plan does not limit the application of ISD. The same principle applies; one must select a relevant point and run the method.

Each histogram can be considered the “signature” of the surface derived from under routine clinical conditions. Nevertheless, the generation of shape histograms from a single point might produce misleading results in extreme scenarios. For example, any distribution rotation around this point or axis will create identical histograms to the original one. The limitation of a mirrored distribution to the reference one is very rare to occur in clinical practice.

However, for all dose distributions under test, four isodoses (50%, 75%, 80%, and 90%) were tested for possible rotations around the isocenter by creating histograms from three randomly selected non‐coplanar points.

In the rare scenario of the evaluation distribution rotation, the shape histograms produced by the isocenter will be identical (Bray‐Curtis metric = 0). However, the shape histograms generated by another point will be different (Bray‐Curtis metric≠0).

**FIGURE A6:**
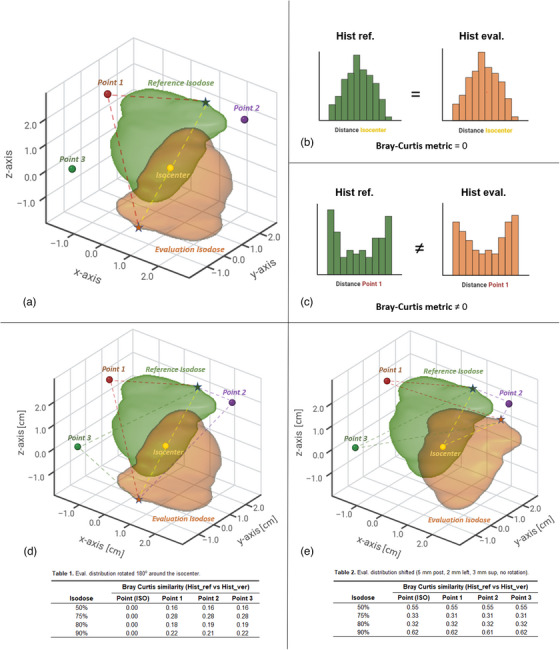
(a) Graphical representation of the mirrored distribution limitation. For simplicity, we present one reference isodose (90%) of a prostate plan (green surface) and its rotated version (180^o^) around the isocenter (orange surface). The distances from the isocenter will be identical for the same point on the surface (green/orange star), while the distances from a random point (Point 1) will be different. (b) Shape histograms (ref. and eval.) generated using the isocenter as a reference point on a rotated distribution. Both histograms will be identical; thus, the Bray‐Curtis (BC) similarity measure will be zero. (c) Shape histograms generated using Point 1 (not a pivot point). The shape histograms will be different, and Bray‐Curtis will have a non‐zero value. (d) Pre‐flight check of ISD method in its current form. On a distribution, we use the isocenter and three non‐coplanar points to compare isodose reference with the evaluation isodose. In the case of rotation, for a given isodose, the BC value will be zero from the isocenter, but all the other points will have a similar non‐zero value (Table 1) When the distributions are not rotated, all points, including the isocenter will generate very similar BC values, as shown in Table 2.

If the Bray‐Curtis result is the same for all four points (including the isocenter) per sampled isodose, then the whole evaluation distribution is not rotated around the isocenter (see Appendix, Figure A6). This step prevents any related introduced errors to the results and the ISD method can be used as described above.

A future investigation will focus on other histogram generation equations, not using the isocenter exclusively, eliminating the possibility of a “mirrored distribution” error. For example, the production of shape histograms using the distances from the isocenter and those from one or more non‐coplanar points will integrate the pre‐flight check for rotations into the histogram generation process—nevertheless, the benefit of over‐ or under‐dosage information that a single point offers should not be compromised.

The shape histograms serve as probability density functions of the points of each isodose. That means that even for sparser dose distributions, a prediction can be made on the distance of the missing points from the isocenter. The latter and the fact that each isodose produces tolerance limits minimizes the need for extensive interpolation of the data.

In the case of PSQA, the ISD method does not “compress” error information to a single value, enabling the user to evaluate the results with a more clinical perspective: the plan can be evaluated after reviewing the entire ISD plot thoroughly, providing an overview of the mismatches on the entire dose range, a unique feature that statistical methods typically lack. The latter is also an advantage compared with DVH‐based plan verification methods,^27^ where only the parts of an isodose included in an OAR‐PTV contour are reported. Nevertheless, the precise position of a spotted mismatch is not reported in the ISD plots. This limitation can be lifted through second‐level processing of the results.

Furthermore, the deployment of all discrepancies per isodose in one graph enables the user to perform the analysis in two regions (“PTV” and “Normal Tissue”). This feature allows the evaluation with individually set tolerance criteria per region (e.g., by applying no symmetrical acceptance limits). For instance, one can apply more tolerant limits for higher doses in the PTV area and lower doses in the rest of the plan.^23^

Tolerance limits for the ISD method are not artificially or empirically generated as symmetric, geometrical boundaries around the points of measurement (ellipses, envelopes).^24^ They are coherently justified by the multileaf collimator (MLC) quality assurance tolerances, and they are linked to the actual capabilities of the linac. For IMRT and VMAT performing linacs, MLC tolerance equals ± 1 mm.^20^, ^21^ Mean isodose distance is a plan dose gradient metric that can detect where the high dose area is located.^19^ The reference isodose ISO_mr_ is selected for each case at the steepest dose gradient of the plot of the isodose differences. Then, the dose difference at ± 1 mm around ISO_mr_ is applied uniformly to all the other isodoses. By applying the MLC criteria to this area, we enable the tolerances to be adapted accordingly to steeper or shallower dose gradient regions. In addition, in the case of multiple‐dose prescription levels, the definition of more than one ISO_mr_ level is possible, indicating the location of each PTV.

The ISD method uses the actual isodoses of the plan to define acceptance limits. This process results in non‐uniform spatial criteria, which are naturally determined by the local dose gradient, individually for each isodose. Figure 3 highlights the importance of this feature. The varying range of distances and the different behavior per plan/treatment site justify the need for individually set acceptance criteria, regionally and per plan. For instance, for the H&N plan, a 2 mm distance criterion around the 65% isodose can imply a 2% difference in dose. However, at 90% isodose, a similar distance could correspond to less than a 1% dose difference. For a different treatment site (e.g., prostate), a 2 mm spatial criterion on the aforementioned isodose levels could result in different dose variances. Setting tolerance limits using the dose gradient of the plan has also been reported in the literature. Jiang et al. used the mean plan gradient to refine tolerances.^25^ However, the ISD method exploits local dose gradients,^8^, ^26^ incorporating the well‐established MLC QA tolerance level.

The presented results revealed that differently distorted plans could result in diverse effects on the final dose delivery, despite having similar gamma passing rates. The geometric parametrization function that ISD uses is susceptible to detecting mismatches since every alteration to the shape of the isodose directly affects the resulting histogram.

A different use of ISD could be the analysis of 2D QA measurements, such as film analysis. In that case, the ISO_mr_ would be 50%, which defines the field edges. In addition, comparing iso‐value surfaces or contours to address differences is broader than the field of radiation therapy. Other scientific fields can also exploit the proposed method to evaluate variations in iso‐valued lines (isoheight, isotherm or isobar maps, isomagnetic charts, isocandela, and isoluminance diagrams).

Although the authors present a specific methodology, this work serves primarily as a proof of concept. Further investigation could be conducted. For example, different histogram‐producing metrics and no symmetrical tolerances could be evaluated using other reference points, offering added perspectives to the ISD method. Future work will additionally include measurements on patient‐specific systems. Finally, the clinical significance and the correlation of ISD results with DVH in the case of PSQA need to be explored.

## 5 CONCLUSION

In conclusion, we developed an ISD method that offers a meaningful, QA‐related, registration‐free, and clinically oriented manner of dose distribution comparison. The full spectrum of the dose levels of a plan can be quantitatively assessed, with high sensitivity. This method can be used either as a standalone or an auxiliary tool to the well‐established evaluation procedures, overcoming significant limitations reported in the literature.

## AUTHOR CONTRIBUTIONS

Stefanos Diamantopoulos, Kalliopi Platoni, Pantelis Karaiskos, Vassilis Kouloulias, and Efstathios Efstathopoulos contributed to study design, data collection and study execution, data analysis and interpretation, and preparation of the manuscript.

## CONFLICT OF INTEREST STATEMENT

The authors declare no conflict of interest.

## 1

Figures a (9.a‐13.a) show the ISD values per isodose for longitudinally inserted shifts (superior direction), Figures b (9.b‐13.b) for vertical shifts (posterior direction), Figures c (9.c13.c) for lateral shifts (Left direction), while Figures d (9.d‐13.d) show arbitrary induced shifts in all directions for each anatomical site.
